# A partial migrant relies upon a range-wide cue set but uses population-specific weighting for migratory timing

**DOI:** 10.1186/s40462-021-00298-y

**Published:** 2021-12-20

**Authors:** Nils Linek, Paweł Brzęk, Phillip Gienapp, M. Teague O’Mara, Ivan Pokrovsky, Andreas Schmidt, J. Ryan Shipley, Jan R. E. Taylor, Juha Tiainen, Tamara Volkmer, Martin Wikelski, Jesko Partecke

**Affiliations:** 1 Max Planck Institute of Animal Behavior, Radolfzell, Germany; 2 Department of Biology, University of Konstanz, Konstanz, Germany; 3 Faculty of Biology, University of Białystok, Białystok, Poland; 4 Michael-Otto-Institut im NABU, Bergeshusen, Germany; 5 Department of Biological Sciences, Southeastern Louisiana University, Hammond, USA; 6 Institute of Plant and Animal Ecology, UB RAS, Ekaterinburg, Russia; 7 Institute of Biological Problems of the North, FEB RAS, Magadan, Russia; 8 Natural Resources Institute Finland, Helsinki, Finland; 9 Lammi Biological Station, University of Helsinki, Lammi, Finland; 10 Centre for the Advanced Study of Collective Behaviour, University of Konstanz, Konstanz, Germany

**Keywords:** Songbird migration, Departure decision, Control mechanisms, Environmental cues

## Abstract

**Background:**

Many birds species range over vast geographic regions and migrate seasonally between their breeding and overwintering sites. Deciding when to depart for migration is one of the most consequential life-history decisions an individual may make. However, it is still not fully understood which environmental cues are used to time the onset of migration and to what extent their relative importance differs across a range of migratory strategies. We focus on departure decisions of a songbird, the Eurasian blackbird *Turdus merula*, in which selected Russian and Polish populations are full migrants which travel relatively long-distances, whereas Finnish and German populations exhibit partial migration with shorter migration distances.

**Methods:**

We used telemetry data from the four populations (610 individuals) to determine which environmental cues individuals from each population use to initiate their autumn migration.

**Results:**

When departing, individuals in all populations selected nights with high atmospheric pressure and minimal cloud cover. Fully migratory populations departed earlier in autumn, at longer day length, at higher ambient temperatures, and during nights with higher relative atmospheric pressure and more supportive winds than partial migrants; however, they did not depart in higher synchrony. Thus, while all studied populations used the same environmental cues, they used population-specific and locally tuned thresholds to determine the day of departure.

**Conclusions:**

Our data support the idea that migratory timing is controlled by general, species-wide mechanisms, but fine-tuned thresholds in response to local conditions.

**Supplementary Information:**

The online version contains supplementary material available at 10.1186/s40462-021-00298-y.

## Background

For migratory species, the timing of annual migration is an integral component of their life history [1]. Although optimal departure for autumn and spring migration is consequential for fitness and survival throughout the year [2, 3], the timing and duration of migration often varies widely among individuals, populations and species [4, 5]. The critical decision when to initiate migration is thought to be based in significant parts on information gathered from the local environment [6–8]. Understanding the mechanisms that underly variation in migration timing and the consequences to individuals survival and fitness is paramount to our understanding of migration as a whole [9, 10].

**Fig. 1:**
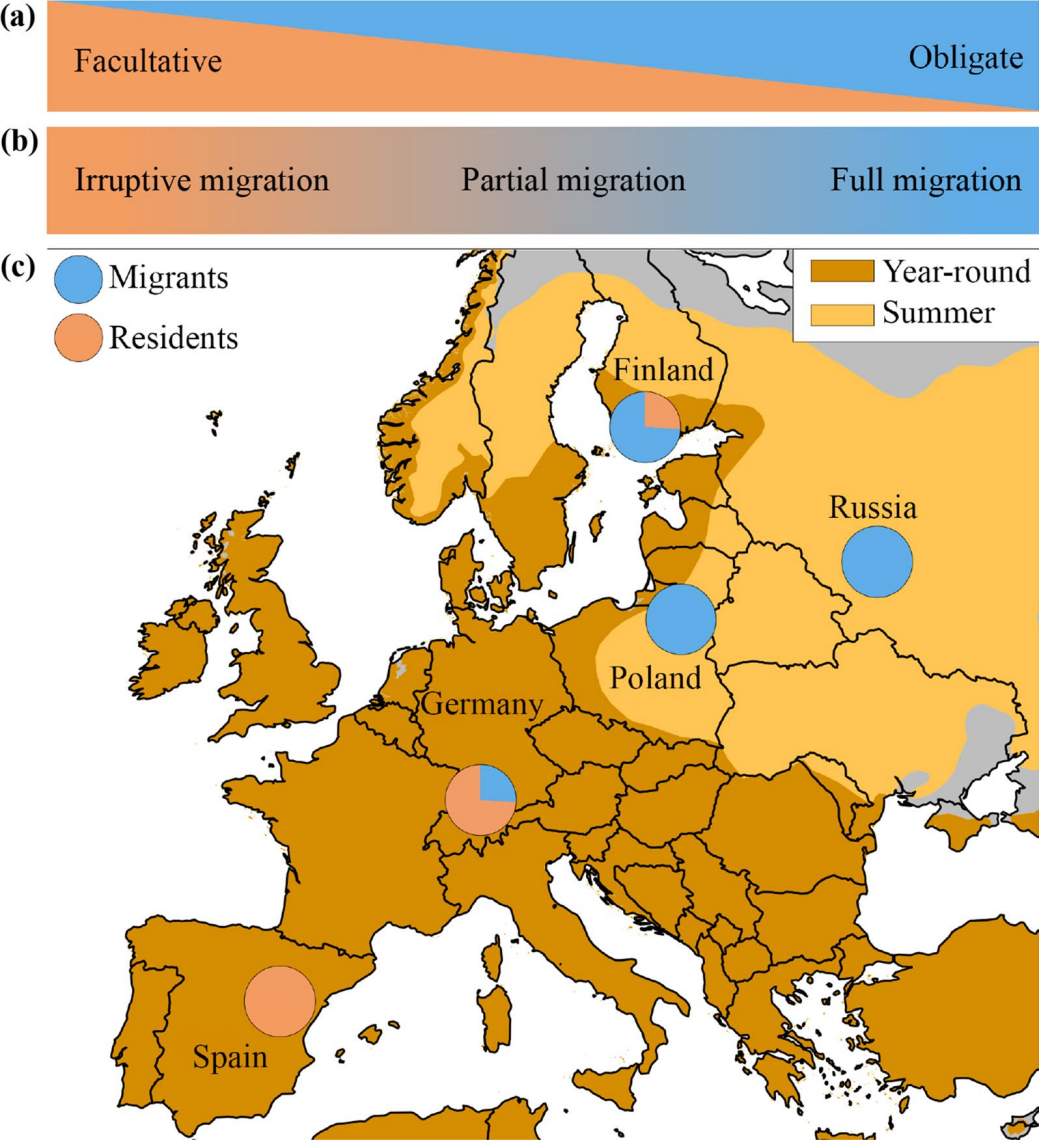
**a** Proximate control mechanisms for migration: Two extremes along a continuous gradient. On one side facultative (environmentally induced) and on the other end, obligate migration (intrinsic control mechanisms). **b** Different migration types (ranging from irruptive to full migration) which are linked to specific proximate control mechanisms (see (**a**)). **c** Locations of study sites for Eurasian blackbirds (*Turdus merula*). While the Spanish population of blackbirds is fully resident, departures from partial (Germany and Finland) and fully (Poland and Russia) migratory populations are used in this study

Migration is a complex life-history stage, and its underlying spatiotemporal organisation is assumed to be part of an endogenous, genetically inherited migration program encoding when, where, and how far to migrate [11–13]. The complexity can be visualised in a continuum between rigid obligate migration and flexible facultative migration [14] (Fig. 1a). Obligate migration is typically characterised by its regularity, consistency, and predictability and is often associated with fully migratory species (such as Common Cuckoos *Curculus canorus*, Red-backed Shrikes *Lanius collurio,* or Thrush Nightingales *Luscinia luscinia* [15]; Fig. 1b). On the other extreme, facultative migrants such as Pine Siskins *Spinus pinus* supposedly base migratory decisions mainly on environmental conditions that, e.g. predict food abundance [16]. This irruptive migration (Fig. 1b) can flexibly respond to changing local conditions. Thus, it is thought to be less regulated by genetic control mechanisms that would be potentially more rigid in their expression.

Independent of where species or populations lie along the migration continuum, individuals rely on environmental cues to assess local proximate information to determine the timing of migration [2]. Environmental cues which contain information about present and future conditions are likely selected based on their historical relationship to fitness [17], but can also vary in their precision and reliability regarding such conditions. Day length, for instance, provides information on coarse long-term trends ranging from weeks to months (e.g., seasonal climate trends) and are used by obligate migrants to time their migration [18, 19]. Migration is initiated either by triggering internal migration programs directly to specific day length thresholds or by calibrating internal clock mechanisms of individuals [18]. On a finer temporal scale in the order of days to weeks, cues such as ambient temperature can provide information about the near future, e.g. upcoming challenges in thermoregulation [20] or information on resource abundance [21], because of the strong influence of temperature on availability of prey insects [22] and worms [23]. Finally, cues, which offer information about the immediate future, such as rapidly-changing atmospheric conditions related to weather (e.g., wind characteristic [24], overcast conditions [25], and atmospheric pressure [26]), likely influence departure decisions in many species and populations. This influence is because of prevailing weather conditions can directly affect immediate energetic costs and survival during migration [25, 27] or in the case of cloud cover, opportunities to use celestial navigation [28, 29].

Although all migratory species likely integrate information from some environmental cues operating at different time scales to determine the optimal timing of departure [30], the exact set of cues used and the magnitude of their effects likely vary between species depending on their migration strategies and even within individuals along their migration routes [31]. A comparison of long- and medium-distance migratory species, for instance, showed that long-distance migrants were least selective at a stopover site for environmental cues such as temperature, atmospheric pressure, and wind conditions compared to medium-distance migratory species [32].

Similar differences in cue selection and responsiveness may also occur between different populations of the same species, which vary in their migration strategies. For example, propensity to migrate often varies across longitudinal, latitudinal, and altitudinal gradients, and wide-ranging species such as the Eurasian blackbird *Turdus merula* often have non-migratory (in southern Europe, i.e. Spain), partially migratory (central Europe and coastal areas), and fully migratory populations in continental northeast Europe (Fig. 1b, c). In general, populations in breeding areas with harsh environmental conditions during the non-breeding season in the northern or eastern parts of the species range exhibit a higher propensity to migrate or full migration. This is accompanied by potential longer migration distances than populations in milder regions with a lower proportion of migrants and shorter migration distances (Fig. 1) [33–36]. In an obligate partial migrant, the blackcap (*Sylvia atricapilla*), propensity to migrate and migration distance (measured as nocturnal activity) are genetically correlated [37]. Due to earlier and potentially faster deterioration of environmental conditions during autumn and longer migration distance, fully migratory populations in the north or east likely face stronger time constraints to initiate their migratory journey. In addition, for obligate migrants, there is likely a stronger selection for reliable accuracy of migration onset regarding favourable flight conditions en route and on stopover sites [38], because of the potential fitness and survival consequences of mistiming in these populations.

In contrast, for partially migratory populations, the selection for accurate timing of migration onset may be relaxed because those breed in areas with milder environmental conditions in the autumn and have potentially shorter migration distances [34]. This potential lack of environmental pressure during winter might also lead to certain risk assessment scenarios where birds decide to overwinter at their breeding site, but if winter conditions become unbearable, they suffer severe fitness consequences or need to perform some form of emergency winter migration [39–41]. Accordingly, partially and fully migratory populations of the same species may feature different decision criteria in evaluating the optimal timing of departure for migration, i.e., they may differ in the use of specific environmental cues for their departure decisions (Fig. 1a, b).

To understand how migratory strategy determines which environmental cues are used to initiate migration departure, we used radio-telemetry to track Eurasian blackbirds from populations exhibiting either partial or full migration. By combining environmental data with individual-level tracking data, we compared migration departures of individuals from four distinct populations along a gradient of migratory strategies (Fig. 1c). We expected migrants from fully migratory populations in Russia and Poland to depart earlier in the season followed by migrants of the partial migrant population in Finland and lastly migrants of the partial migrant population in Germany.

We tested the hypothesis that birds from fully migratory populations which breed in areas characterised by harsh environmental winter conditions will base their departure decisions on cues that are more predictable across years and reliable for far-away overwintering sites, such as day length, and less upon more flexible environmental cues such as ambient temperature. We predicted this would result in a more synchronised and rigid timing of migration among individuals and years. In contrast, individuals from partially migratory populations that likely migrate shorter distances and where some part of the population remains resident year-round may use cues containing more immediate information about local environmental conditions such as immediate changes in the ambient temperature. Individual flexibility, which leads to a greater population-level variation in departure dates, should result in less synchrony of departures.

As a consequence of differences in departure timing of migrants originating from fully and partially migratory populations, we examined the hypothesis of whether migrating individuals of different populations use a similar, species-wide set of cues with either identical or population-specific threshold values.

We further predicted that favourable weather conditions should affect both fully and partially migratory populations similarly because the decision to migrate should follow the same general principles of minimised survival risk and energetic costs during flight independent of the migration strategy. However, due to differences in time constraints and consequences of delayed onset of migration, we predicted that departure decisions of individuals of fully migratory populations should be less affected by ephemeral weather conditions than those of partially migratory populations.

## Methods

### Study sites and species

We monitored 610 Eurasian blackbirds (*Turdus merula*) at four geographically distant study sites (Fig. 1): The sites with partially migratory populations included southern Germany (47.7801° N, 9.0203° E, 651 a.m.s.l.) where we monitored 527 blackbirds (291 males, 207 females and 29 birds of unknown sex during nine field seasons from 2009 to 2017) and southern Finland (61.0975° N, 25.1579° E, 154 a.m.s.l.) where we monitored 34 individuals in three field seasons from 2014 to 2016 (21 males, 13 females). Both regions consist of mixed deciduous and coniferous forests and are characterised by large bodies of water, which moderate the climate towards milder temperatures. However, during winter (2009–2017 during December and January), the Finnish study site has, on average colder temperatures (− 6.1 °C) compared to the German site (− 3.8 °C). Fully migratory populations were monitored in Russia (55.4582° N, 37.1791° E, 182 a.m.s.l.) where we radio-tracked 30 blackbirds (23 males, seven females during three field seasons: 2014–2016) and in north-east Poland (53.3483° N, 22.5927° E, 106 a.m.s.l.) where 19 individuals were studied (15 males and four females during two field seasons: 2015–2016). Those regions can be defined as continental with cold winters (average temperature for December and January in Russia: − 9 °C, Poland: − 4.9 °C), and they consist primarily of coniferous forests. At all study sites, we captured birds from May until August using mist nets. Based on previous ring recoveries during winter, we expected migratory birds from all sites to leave in a southwestern direction with the two fully migratory populations (Russia and Poland) covering longer migration distances [33–36]. Both adults and immatures were included. The age and sex of captured birds were determined based on differences in the colouration of plumage and beak [42] (Additional file 1: Table S1).

### Capture and tracking methods

After capture, each bird was fitted with an aluminium band with a unique ID and a backpack with a radio transmitter (≤ 2.6 g produced either by 1. Sparrow Systems, Fisher, IL, USA (2009–2012, 2014–2015), 2. the Swiss Ornithological Institute, Sempach, Switzerland (2014), or 3. Holohil Systems Ltd., Canada (2013)) via a leg-loop harness. All radio transmitters have sent a radio impulse every 1.5 to 3 s from deployment until at least next spring/summer. The mean weight of the captured blackbirds was 88 g resulting in an additional load of 3% with the radio transmitter, which has a battery life of at least nine months. To determine the status (presence/absence and alive/dead), non-breeding strategy (migrant versus resident) and the timing of departures of individuals, we deployed one to five automated receiver units (ARU, Sparrow Systems, Fisher, IL, USA) at haphazard locations on each study site [43]. Each ARU searched for up to 60 selected frequencies within a maximum timeframe of 240 s. ARUs were connected to H-antennas (ATS, Isanti, MN, USA), mounted at the height of 3 to 12 m. 24-h ARU monitoring enabled us to precisely determine departure events via a rapid decline of signal strength of the radio transmitters. We used ARU data sightings and manual tracking to ensure the absence of an individual within a 2.5 km radius. Manual tracking was done via a combination of handheld H antenna (Andreas Wagener Telemetry Systems, Köln, DE) and Yaesu VR 500 receiver (Vertex Standard USA, Cypress, CA, USA). For the German population, we also used car-mounted Yagi-antenna (AF Antronics, Inc., Urbana, IL, USA) and an airplane equipped with two H-antennas and two Biotrack receivers (Lotek, Newmarket, ON, Can) to ensure departure of an individual within a 20 km radius of the study site and to validate the 2.5 km radius, which we used to define departures in the other study areas. For seven German birds (one in 2009, 2010 and five in 2015), the departure could not be identified on an ARU recordings, and the departure date was calculated subsequently as the mean between the last time the bird was tracked and the first date missing. All post-breeding departures between the 2nd of September and the 24th of November were included in our analysis. Later departures occurred only in the German population and have been phenotypically different based on previous studies [39]. Hence they were classified as winter migration or irruptive migration events. This study also included published data from our German study site collected before 2016 [39, 44].

### Environmental variables

We selected day length as a possible cue since photoperiod has known biological significance in controlling timing for migration [45]. Day length was calculated in hours per day via the *maptools* package using R statistical software V. 3.3.2 [46, 47]. We included local ambient temperature at 2 m above ground in our model as the ambient temperature indicates future energetic costs due to thermoregulation [20]. The temperature may also provide information about the availability of food resources in the near future [48]. Because ambient temperature is highly correlated with day length and will be colder as the season progresses during autumn, we calculated the residuals of temperature each night out of a linear regression of date and temperature, defined as residual temperature. This calculation allowed us to measure the deviation from the expected temperature that would historically occur at this point of time within a year and is perceived as colder or warmer on average. Since cloud cover strongly impacts navigation at night [28], we also included the percentage of cloud cover in our model. To represent essential characteristics of flight conditions, we used atmospheric pressure (at surface level), wind direction and speed (at surface level) and used this to calculate likely tailwind assistance and crosswind in meters per second. These cues likely provide immediate information about upcoming weather conditions in the subsequent hours and days [26, 49].

The ambient temperature, atmospheric pressure, cloud cover and wind conditions used in the analyses were interpolated from the nearest weather stations in each location by the National Centers for Environmental Prediction [50]. The data were derived for the four breeding locations in a 6-h resolution, using the *RNCEP* package [51], and a mean for each night between 6 and 12 pm was calculated. Due to the fact that blackbirds leave the ARU signal range relatively quickly with their departure, unfortunately, no statements can be made about the behaviour and environmental conditions after initial departure. Therefore we estimated the same mean south-west migration direction for all four populations [52] based on ring recoveries of blackbirds. Tailwind assistance was calculated for a species mean departure direction of 225° by *tailwind assistance* = *windspeed* ∗ cos(*wind dir*e*ction* − *mean departure direction*). Crosswinds perpendicular to the assumed mean departure direction have been calculated by crosswinds = *windspeed* ∗ sin(*wind dir*e*ction* − *mean departure direction* [53].

### Data analysis

Statistical analyses were performed using R statistical software V. 3.3.2 [46].

To account for the unbalanced sample sizes between the sites, we also performed the following analysis with a balanced sample size in all populations, by reducing the German data set to the same years as the other sampled locations. This analysis provided similar results with the same order of importance in the predictors.

In order to compare timing and departure conditions between populations and migration types, the equality of variances between different sites and their parameters were ensured using Levene’s test [54] before running the corresponding linear models with site and migration type as explanatory variables in separate models (package *stats*). Least-squared means post-hoc tests were performed when sites or migration types differed in the conditions during their departure nights. *P*-values of multiple comparisons were adjusted via the Benjamini–Hochberg method (package *emmeans*) [55]. Since the normalised mean atmospheric pressure and mean wind result in a measured value of zero, a one-sample t-test was used to test for a general preference of these environmental conditions at departure days across all populations. Variances in departure timing between populations were compared using Bartlett’s test of homogeneity of variances (package *stats*) [56] while we adopted a 5% significance threshold for all models.

After testing for occurring differences in departure conditions between populations, we investigated the potential influence of these environmental variables on individual departure decisions. Therefore, we used a time-dependent Cox proportional hazard model implemented in the *survival* package [57]. The Cox proportional hazard model describes the probability of departure over time as a function of a baseline probability which can be modified by fixed variables like population or time-varying explanatory variables like weather conditions and day length [58]. All weather parameters were scaled by subtracting the mean and dividing it by the standard deviation for each population and year to make their effect sizes comparable within and between each population within the model. This was also done to account for general differences between the microhabitats and annual variation as study site-specific differences in meteorological conditions are corrected for in scaled variables which allows to only focus on perceived changes in each population. However, we scaled day length only within each site but across all years, as day length over time does not vary between years. We included the sex, age and unique ID of the birds in the model, but it had no significant influence on the outcome and was therefore omitted.

We restricted the analysis to a timeframe of 47 days before the first migratory departure in each year and site, as 47 days is the mean migration window across all four populations based on our radio-telemetry measures (Additional file 1: Table S2). We used an information-theoretic approach for model selection from a global starting model to calculate all possible models via the “dredge” function (package *MuMin*) [59] (i.e., all combinations of including or excluding each variable and its interaction with populations). To evaluate the likelihood of a model being ‘best’ among all other candidate models and estimate their relative predictor importance [60], we ranked them based on their Akaike weights derived from differences of their Akaike information criterion (AIC) corrected for a small sample size, AICc [61]. Out of all model combinations, we selected those within a ΔAICc < 2 [62] from the top model, which included those with strong to moderate support (Additional file 1: Table S3).

The estimated parameters for all predictors included in these top models were then calculated by model averaging [63] with the “model.avg” function (package *MuMin*) [59]. Predictors of the global model but with no meaningful influence were not included in the top model subset. Least-squared means post-hoc tests on the most complex of the candidate models were performed when the averaged model results showed a significant interaction of environmental cues and populations. P-values of multiple comparisons were adjusted via the Benjamini–Hochberg method (package *emmeans*) [55].

Finally, for the Cox proportional hazard model, we ran a Schoenfeld test (cox.zph function, package *survival*) [57] to ensure the model assumption are met and further double-checked for correlations between all used variables within the timeframe of analysis (Additional file 1: Table S4).

## Results

### Proportions of migrants

In total, we observed 212 departures during autumn across all four populations (Additional file 1: Table S1). In Germany, 136 birds of the population migrated, while 391 remained at the breeding site. As expected, the German population can be defined as a partially migratory population, with 26% of birds migrating on average. Our radio telemetry data also verified that the Finnish blackbird population is partially migratory. Out of the 34 monitored birds, we observed 27 migrants, resulting in 74% migrants at the Finnish study site. In Russia (30 migrants) and Poland (19 migrants), no resident individuals were observed, and thus these populations are defined as fully migratory.

### Departure timing during autumn migration

Our long-term monitoring revealed that all departures for autumn migration happened after sunset during the night (mean: 21:40, range: 18:30 to 02:00 UTC).

**Fig. 2:**
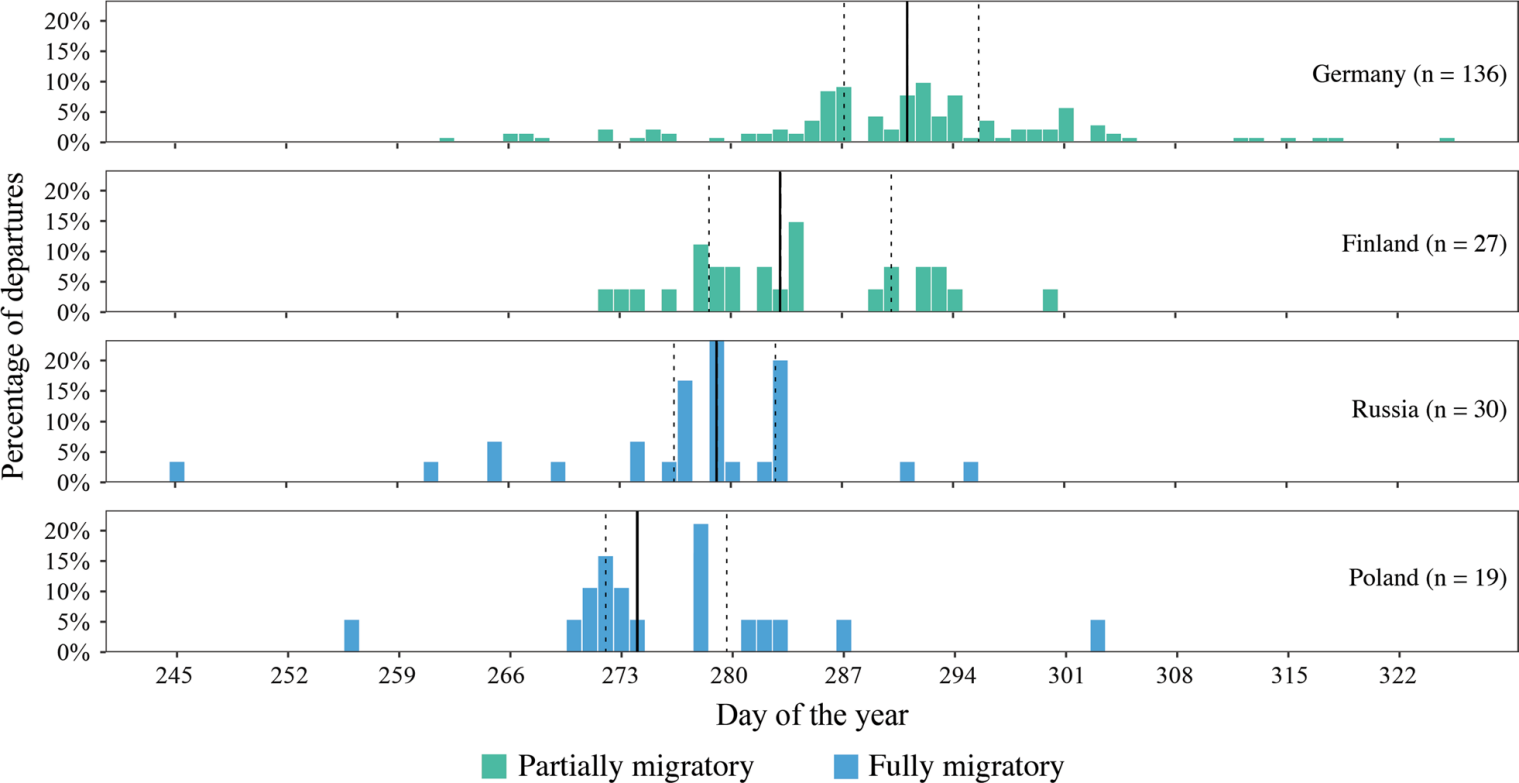
Timing of autumn migration events across all years for each population. The solid black line marks the population median date for departure, while the dashed lines left and right mark the corresponding first and third quantiles. Day 1 = equals 1st. January

On a night-to-night basis, blackbirds from the Russian and Polish populations departed the earliest in the year, followed by the Finnish and later the German populations [F_3,215_ = 26.88, *p* < 0.01] (Fig. 2). By pooling both the two fully migratory (Russian and Polish) and the two partially migratory (Finnish and German) populations, the two migratory strategies (fully versus partially migratory) differed in the timing of the night-to-night departure decision [F_1,217_ = 64.75, *p* < 0.01]. Fully migratory populations left their breeding site 13 ± 2 days earlier than the partially migratory populations (Fig. 2). However, the length of the window when migrants departed (= variance in departure dates) did not differ among the four populations [df = 3, k = 4.18, *p* > 0.24] or the two migration types [df = 1, k = 0.75, *p* > 0.38] (Additional file 1: Table S2).

### Environmental conditions during departures

**Fig. 3:**
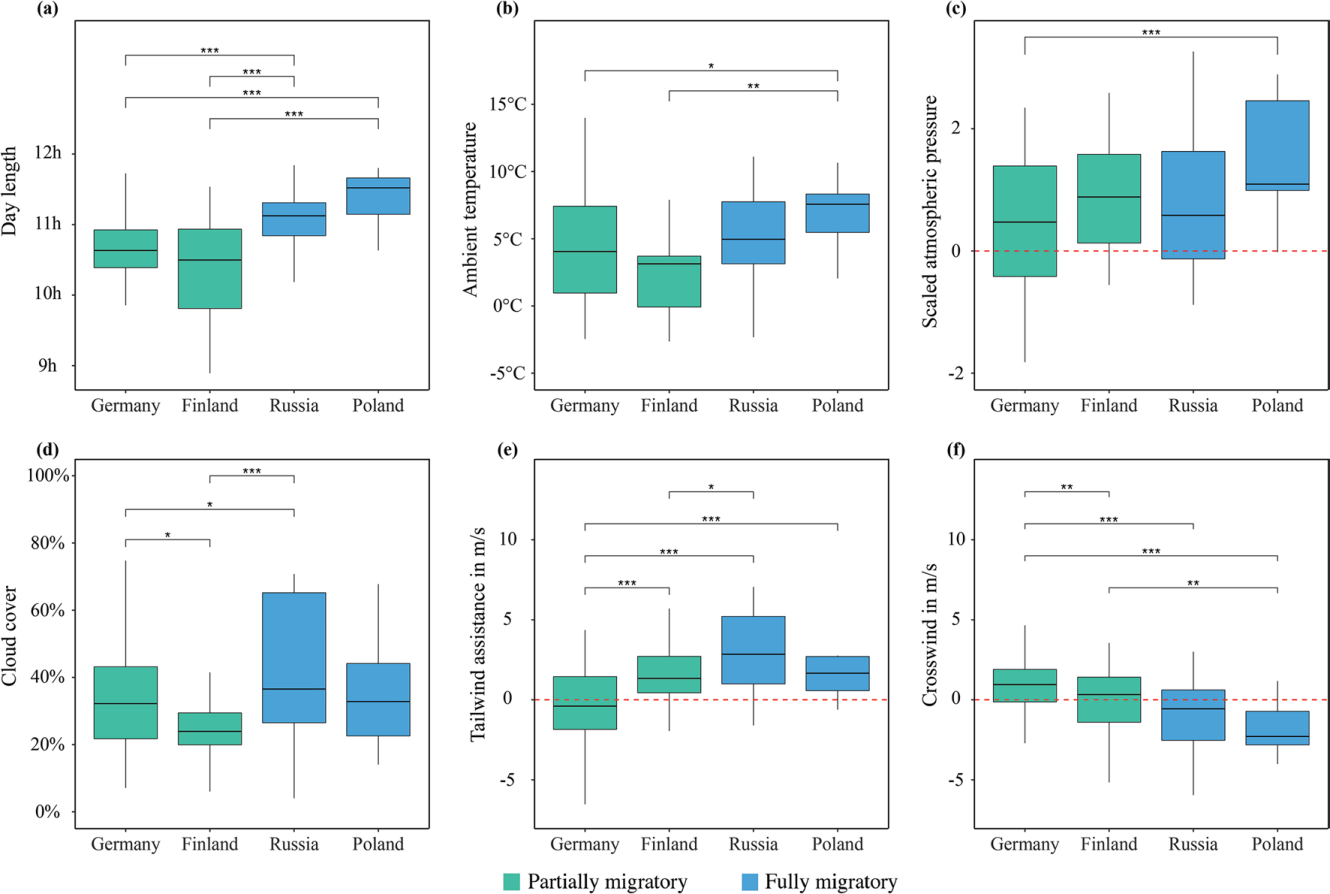
Comparison of day length (**a**), ambient temperature (**b**), scaled atmospheric pressure (**c**), cloud cover (**d**), tailwind assistance (**e**) and crosswind (**f**) at departure nights during autumn migration between blackbird populations. Black solid lines represent the median, boxes represent first and third quartiles, and whiskers describe the 95% confidence intervals. The dashed red line in (**c**) represents mean atmospheric pressure during the entire departure window, while it marks the absence of tail and crosswinds in (**e**) and (**f**). Populations that were identified as different via post hoc tests are joined by brackets noted with the corresponding significant levels (* ≤ 0.05, ** ≤ 0.01, ***≤  0.001)

While no general differences in environmental conditions were found between study sites throughout the overall migration window (i.e. similar temperature ranges, wind conditions etc.) (Additional file 1: Figure S1a–S1f), comparisons of environmental conditions during nights with migratory departures between populations showed significant differences (Fig. 3a–f) and pronounced variance (Additional file 1: Table S5).

#### Day length

Day length at departure differed among the four populations [F_3,215_ = 16.68, *p* < 0.01]. Post-hoc tests indicated that these differences were most pronounced between the two migration types (partial vs. full migration) [F_1,217_ = 45.70, *p* < 0.01], with 0.7 ± 0.1 h longer daylength at departure dates for full than partial migrants (Fig. 3a).

#### Ambient temperature

Populations also departed at different ambient temperatures [F_3,215_ = 4.23, *p* < 0.006]. Blackbirds of the Polish study site departed at warmer temperatures (mean ± standard error: 7 ± 0.9 °C) than conspecifics at the Finnish (2.6 ± 0.8 °C) [β = 4.4, SE = 1.2, t = 3.6, *p* < 0.01] and German study sites (4.3 ± 0.3 °C) [β = 2.7, SE = 1.0, t = 2.7, *p* = 0.02]. However, the temperature (4.4 ± 0.7 °C) during departures of blackbirds at the Russian study site could not be found to be different compared to the other populations. When comparing the two migration types, blackbirds from fully migratory populations departed when it was 1.4 ± 0.7 °C warmer than blackbirds from partially migratory populations [F_1,217_ = 4.31, *p* = 0.04] (Fig. 3b).

#### Atmospheric pressure

The four populations also differed in the relative atmospheric pressure during the nights of departures [F_3,215_ = 6.33, *p* < 0.01]. Relative atmospheric pressure was higher at departures of blackbirds from the Polish study site compared to conspecifics of the German site study site [β = 1.2, SE = 0.3, t = 4.0, *p* < 0.01]. Fully migratory populations departed on nights with higher relative atmospheric pressure than partially migratory populations [β = 0.66, SE = 0.2, t = 3.3, *p* < 0.01]. Across all populations, the atmospheric pressure was higher on nights when birds departed than on nights when birds did not depart [M = 0.56 ± 0.17, t(218) = 6.47, *p* < 0.01] (Fig. 3c).

#### Cloud cover

All populations favoured nights with a cloud cover of less than 50% for their departures [M = 33.36 ± 2.21, t(218) = − 14.84, *p* < 0.01]. However, populations also differed in actual cloud cover during departure nights [F_3,215_ = 5.57, *p* < 0.01]. Departures at the Finnish study site occurred during nights with significant lower cloud cover (23.8 ± 3.1%) compared to those at the German (33.5 ± 1.4%) [β = − 9.8, SE = 3.4, t = − 2.9, *p* = 0.01] and Russian study sites (41.2 ± 2.9%) [β = − 17.4, SE = 4.3, t = − 4.1, *p* < 0.01] (Fig. 3d).

#### Tailwind assistance

In general, tailwind assistance on departure nights differed among populations [F_3,215_ = 19.47, *p* < 0.01]. Tailwind on departure nights at the German study site (− 0.3 ± 0.2 m/s) was lower than at Russian (3.0 ± 0.5 m/s) [β = − 3.3, SE = 0.5, t = − 6.6, *p* < 0.01], Polish (2.2 ± 0.6 m/s) [β = − 2.5, SE = 0.6, t = − 4.1, *p* < 0.01] and Finnish (1.6 ± 0.5 m/s) [β = − 1.9, SE = 5.3, t = − 3.5, *p* < 0.01] sites. Departures from the Russian site occurred during the strongest tailwinds (up to 7 m/s) across all populations, even stronger than during departures at the Finnish site [β = 1.4, SE = 0.7, t = 2.1, *p* < 0.05]. Blackbirds from partially migratory populations left with lower tailwind assistance than those from fully migratory populations [F_1,217_ = 42.16, *p* < 0.01]. However, they did not differ from zero [M = − 0.004 ± 0.39, t(169) = − 0.02, *p* = 0.98]. Fully migratory populations, in contrast, preferred nights where tailwind assistance in their approximated direction of migration was clearly present [M = 2.71 ± 0.77, t(48) = 7.11, *p* < 0.01] (Fig. 3e).

#### Crosswinds

Departures from all four populations differed in their crosswind conditions [F_3,215_ = 18.03, *p* < 0.01] (Fig. 3f). During departures at the German study site crosswinds are mainly towards north-west [M = 0.99 ± 0.25, t(142) = 7.76, *p* < 0.01] and the strongest in this direction compared to all other populations (Finland: [β = 1.06, SE = 0.38, t = 2.79, *p* < 0.01], Russia: [β = 1.7, SE = 0.36, t = 4.69, *p* < 0.01] and Poland: [β = 2.7, SE = 0.44, t = 6.1, *p* < 0.01]). However, while being different between sites, crosswinds could not be found to be different from zero at the study sites in Finland [M = − 0.07 ± 0.85, t(26) = − 0.16, *p* = 0.87] and Russia [M = − 0.71 ± 1.02, t(29) = − 1.43, *p* = 0.16]. In contrast, blackbirds at the Polish study site selected nights for departure with crosswinds primarily towards south-east [M = − 1.71 ± 0.69, t(18) = − 5.19, *p* < 0.01] and also differed significantly from crosswind conditions during departure nights at the Finnish study site [β = − 1.64, SE = 0.54, t = − 3.02, *p* < 0.01].

### Used cues for individual timing of migratory departures

The best averaged Cox proportional hazard model showed that all investigated environmental cues, day length [sum of model weight = 1, *p* < 0.01], residual ambient temperature [sum of model weight = 0.73, *p* = 0.05], atmospheric pressure [sum of model weight = 1, *p* = 0.01] and cloud cover [sum of model weight = 1, *p* < 0.01] as well as tail [sum of model weight = 1, *p* = 0.02] and crosswind affected [sum of model weight = 1, *p* < 0.01] the decision of Russian, Polish, Finnish and German populations to depart during autumn migration (Table 1). Across sites, day length was the most influential predictor, with shorter day lengths increasing departure probability [β = − 3.65, SE = 1.17], followed by the various cues describing flight conditions. In those high atmospheric pressure [β = 0.30, SE = 0.12], increased tailwind assistance [β = 0.29, SE = 0.13] and decreased crosswinds [β = 0.59, SE = 0.11] had a positive effect on the departure probability. Cloud cover instead had a negative effect with obstructed skies reducing the departure probability of blackbirds [β = − 0.42, SE = 0.11]. The overall residual temperature was the predictor with the smallest but still significant effect. Colder than normal temperatures increased departure probabilities across all populations [β = − 0.17, SE = 0.09].

**Table 1:** Averaged model for the effects of weather variables and population on departure probability of individual blackbirds

Parameter	Estimate (*β*) ± SE	95% CI	*p*-value
Day length	− 3.65 ± 1.17	− 5.95 ± − 1.35	** < 0.01**
Residual ambient temperature	− 0.17 ± 0.09	− 0.34 ± 0.01	**0.05**
Atmospheric pressure	0.30 ± 0.12	0.06 ± 0.53	**0.01**
Cloud cover	− 0.42 ± 0.11	− 0.64 ± − 0.21	** < 0.01**
Tailwind assistance	0.29 ± 0.13	0.04 ± 0.53	**0.02**
Crosswind	0.59 ± 0.11	0.38 ± 0.81	** < 0.01**
Population (Finland) × Day length	0.73 ± 0.54	− 0.33 ± 1.79	0.18
Population (Russia) × Day length	1.95 ± 0.81	0.36 ± 3.54	**0.02**
Population (Poland) × Day length	1.82 ± 0.64	0.56 ± 3.08	** < 0.01**
Population (Finland) × Atmospheric pressure	− 0.27 ± 0.37	− 0.99 ± 0.45	0.46
Population (Russia) × Atmospheric pressure	− 0.36 ± 0.37	− 1.08 ± 0.36	0.33
Population (Poland) × Atmospheric pressure	1.00 ± 0.40	0.21 ± 1.79	**0.01**
Population (Finland) × Cloud cover	0.38 ± 0.44	− 0.48 ± 1.24	0.39
Population (Russia) × Cloud cover	− 0.74 ± 0.31	− 1.33 ± − 0.14	**0.02**
Population (Poland) × Cloud cover	− 0.29 ± 0.46	− 1.19 ± 0.60	0.52
Population (Finland) × Tailwind assistance	0.37 ± 0.28	− 0.17 ± 0.92	0.18
Population (Russia) × Tailwind assistance	1.31 ± 0.44	0.44 ± 2.17	** < 0.01**
Population (Poland) × Tailwind assistance	0.11 ± 0.27	− 0.42 ± 0.64	0.68
Population (Finland) × Crosswind	− 0.63 ± 0.25	− 1.12 ± − 0.14	**0.01**
Population (Russia) × Crosswind	− 0.29 ± 0.39	− 1.05 ± 0.47	0.45
Population (Poland) × Crosswind	− 1.28 ± 0.30	− 1.86 ± − 0.69	** < 0.01**

Adjusted Akaike’s Information Criterion (AICc) has been used to determine the final candidate models (Additional file 1: Table S1)

Average model estimates, adjusted standard errors (SE), 95% confidence intervals (CIs) and associated *p*-values of parameters included in the candidate models. *p*-values ≤ 0.05 are given in bold font. The reference category for species is the German population

The best averaged cox proportional hazard model also revealed significant two-way interactions between populations and timing-relevant cues (Table 1). This suggests population-specific differences in the magnitude of influence on departure decision probabilities. Post-hoc tests on the interaction between populations and day length indicated that departure probability of blackbirds from both fully migratory (Russia and Poland) populations was less strongly affected by decreasing day length than that of migrants of the German and Finnish partially migratory study populations [β = 0.69, SE = 0.33, t = 2.09, *p* = 0.04]. The significant two-way interaction between populations and atmospheric pressure indicated that blackbirds of the Polish study site had a higher departure probability with higher relative atmospheric pressure compared to migrants of the German study site [β = 1, *p* = 0.01] (Table 1), and post-hoc tests revealed that the influence of atmospheric pressure was also greater compared to the Russian migrants [*p* = 0.02] (Additional file 1: Table S6). In Russian migrants, the significant two-way interaction between species and cloud cover indicated that they had been more influenced in their departure probability by cloud cover compared to migrants of the German partially migratory population [β = − 0.74, *p* = 0.02] (Table 1). Also, the significant two-way interaction between the Russian population and tailwind assistance together with post-hoc tests indicated that Russian migrants had a higher departure probability [β = 1.36, SE = 0.40, *p* < 0.01] when tailwind assistance was stronger compared to all other migrants (Table 1, Additional file 1: Table S6). A significant interaction between the Finnish and Polish populations with crosswind suggested that German migrants have been influenced to a greater extent by winds from the southeast than migrants from the Finnish [β = − 0.63, *p* = 0.01] and Polish [β = − 1.28, *p* < 0.01] populations (Table 1). Observed Polish migrants, on the other hand, had a higher departure probability with stronger crosswinds from the northwest compared to migrants from Germany [β = 1.29, *p* < 0.01] and migrants from the Russia site [β = 1.15, *p* = 0.02] (Additional file 1: Table S6).

## Discussion

By combining environmental data and radio tracking of Eurasian blackbirds from four populations with different migration strategies, we identified a common species-wide cue set used for the decision of migratory departures, but also confirmed variation in migratory timing itself and revealed effects of cues that differed in their magnitude between populations exhibiting different migration strategies.

Fully migratory populations from Russia and Poland departed earlier in the year than their partial migrant conspecifics in Finland and Germany. This result is consistent with previous studies [40], and we hypothesise that several factors like earlier deteriorating environmental conditions at the breeding site, relatively longer migration distances and thus longer migration drive earlier departures in Russia and Poland [52]. Considering the potentially greater consequences of mistiming in migrants, which migrate longer distances, we predicted higher synchrony in departure dates as they might be under stronger time constraints during migration. However, our results did not support this idea, as we found no significant difference in the variation of departures between partial (relatively shorter migration distances) and full migrants (relatively longer migration distances). While time constraints have been shown for spring migration to secure high-quality breeding territories [9, 64] or reproductive partners [65, 66], our results about initial departure conditions during autumn migration are not consistent with previous assumptions [67]. We suggest that also during the typically slower and extended autumn migration [68], fully migratory populations of blackbirds may not be under stronger selection for optimal departure timing to leave their breeding sites than their partial migrant conspecifics. However, as we only recorded the initial departure, we cannot draw any conclusions about time constraints en-route, especially since stopover locations and durations remain unknown.

The environmental conditions studied had been similar across study sites during the migration window and spanned the same ranges (Fig. 3; Additional file 1: Figure S1a-S1f). However, they differed during nights with departures. This would suggest that local differences in the investigated environmental factors did not cause the timing differences between populations. Hence there did not appear to be a uniform species-wide threshold for the cues blackbirds use for departure decisions across their breeding range (Fig. 3). Similar to obligate and potentially long-distance migrants, the fully migratory blackbird populations left earlier in the year at longer day lengths (Fig. 3a) and higher ambient temperatures (Fig. 3b). Therefore, ambient conditions were significantly milder than conditions preceding departures in partially migratory populations. This finding, in combination with the finding that all sites had similar environmental conditions throughout the migration window (Additional file 1: Figure S1a–S1f), supports the idea that anticipated resources at the wintering destination [18] or longer flight distances with optimal flight conditions may inform departure decisions of obligate full migrants [69].

Migrants of all populations appeared to depart during periods of high relative atmospheric pressure, which is a signal for favourable flight conditions [26] already found as an important cue for migratory timing [49]. In particular, the departures of blackbirds at the Polish study site occurred during nights with particularly high atmospheric pressures (Fig. 3c), pointing towards a higher selectivity for such nights. Cloud cover during departures was 33 ± 2% across all populations and showed a general preference for departures at nights with little cloud cover. Clear skies likely facilitate celestial navigation [28, 29] which is thought to be of critical importance for migration in many species [70, 71].

Tailwind assistance and crosswinds at departure nights showed distinct population-specific differences along a geographic pattern. While the investigated north-eastern Russian and Polish migrants departed with more tailwind assistance than the migrants from Germany and Finland, the departures in the German population did not occur during nights with either clear head or tailwind. However, especially the blackbirds from the Russian study site migrated on nights when high tailwind assistance was likely to reduce the energetic cost of migration [72]. The contrasts in crosswind conditions on departure nights between populations are particularly evident in the most south-westerly population of German birds, which departed more often with winds coming from the southeast, and the Polish birds, which started their migration mainly on days with predominantly wind coming from the northwest.

Based on Cox proportional hazard models, all variables, i.e. day length, residual temperature, atmospheric pressure, cloud cover, and wind conditions, significantly influenced the night-to-night probability of departure decision in all blackbirds regardless of their migratory propensity (Table 1). Decreasing day length was the most important factor for night-to-night departure decisions of all studied populations, followed by cloud cover, atmospheric pressure, tailwind, crosswind and residual temperature.

As in long-distance migratory species, decreasing day length seems to be also an important cue for departure decisions in the partial migrant blackbird, a short to medium distance migratory species, during autumn migration. For example, (1) peak autumn migration season across Europe in Blackbirds is during October [52] and individuals do not migrate before their specific migration window even when they experience suitable flight conditions (e.g. high atmospheric pressure and adequate wind conditions) and (2) captive blackbirds in a temperature-controlled common garden experiment exhibited nocturnal restlessness during October as well [73].

However, the likely fine-tuning of the timing of departure on a night-to-night basis appears to be more modulated via imminent flight conditions like wind characteristics and atmospheric pressure, predicting potentially unfavourable flight conditions [27], while also preferring clear skies, which might be necessary for navigation [29]. As such, flight conditions were the second most important factors across all populations (Table 1). These conditions likewise stood out in the first part of our analysis, where all populations showed a strong preference for days with below-average cloud cover, high atmospheric pressure, and population-specific wind conditions compared to residual temperature (Fig. 3). Those conditions are generally characterised by no rain and favourable flight conditions.

Of the environmental factors we studied, temperature was the factor that had the least influence on departure probability. However, in our full and partial migrant populations (residual) temperatures lower than average increased the probability for departure decisions as they likely contain local environmental information about diminishing food resources and increasing thermoregulatory costs [74] (Table 1).

However, the magnitude of the effects of all cues, except residual temperature, differed between full and partial migrant populations. While day length is still the strongest predictor for departure timing for full migrants in Russia and Poland, it was less influential in departure decisions than for partial migrants from Finland and Germany. This appears to contradict our general understanding of how day length influences the seasonal organisation of migration [32, 75] and our expectation that obligate full migrants rely more strongly on predictive cues like day length than facultative partial migrants. One possible explanation for this finding could be that the effects of daylength are masked by other more relevant factors such as appropriate weather conditions essential for every migratory flight. Migrant blackbirds from Russia and Poland, which likely travel longer distances, in particular, may be naturally subject to significantly more energetic constraints and higher fitness consequences [76, 77] compared to migrants from Finland and Germany. They may be more sensitive to the costs of flight energetics [78, 79] and hence are more prone to select nights with optimal flight conditions, which are characterised by high atmospheric pressure and best possible wind support to a greater extent for departure and migration (Table 1, Fig. 3).

In contrast, there is likely a smaller need for optimal flight conditions in short-distance migrants, such as the blackbirds from the German population, as they only travel about 1–3 days to their wintering areas (*Linek *et al*., 2022, in prep*). This hypothesis is supported by two-way interactions between populations and several weather variables (Table 1). Full migrants from Poland had a higher probability for departure at nights with higher atmospheric pressure and with a stronger crosswind from the northwest compared to partial migrants in Germany. Similarly, full migrants from the Russian study side had a higher probability of departure at nights with clearer skies and stronger tailwind assistance than partial migrants. Additional data are necessary to verify this hypothesis. The greater preference of full migrants for particularly good flight conditions and the fact that we could not observe a hard-wired threshold for specific day lengths [80] across populations is consistent with the fact that we did not find greater synchrony in departure dates and highlights the population-specific weighting of environmental conditions for migratory departures.

However, the overall importance of all six environmental variables across all populations suggests that partial migrants from our German and Finnish study sites behave similarly to the other two fully migratory populations by selecting suitable flight conditions for their departures (Fig. 1c, Table 1). Previous research supports our finding that crosswinds and tailwind assistance seem generally important [32, 67]. The combination of lateral drift *en route* and increased energy expenditure imposed by crosswinds [81] seems crucial for all populations and may outweigh the potential time constraints [82] faced by relatively early-departing populations in Russia and Poland.

## Conclusion

In summary, despite discovering that migrants of all populations appeared to depart during favourable weather conditions, suggesting that they are sensitive to the costs of flight energetics [83] and available visual cues [84], we found large differences not only in departure timing between populations and migration types (full and partial migrants) but also in the environmental conditions at their respective departures. These population-specific differences in day length, ambient temperature, atmospheric pressure, and wind characteristics likely indicate local adjustments to their specific environment. In combination, the investigated cues allow initiation, fine-tuning and adjustments to short term weather conditions within a broader migration window, but blackbirds from different populations appear to use population-specific local cue thresholds and weightings when integrating environmental information to initiate migration decisions (also see [85]).

All the cues tested in our study are also correlated to some extent to other environmental conditions and thus may serve as a proxy for other still unknown cues which birds use from their environment to time their migration optimally [2]. In this respect, factors such as food availability, which so far could not be adequately measured for our populations, but likely varies between study sites and influences overall body conditions [86], could be of particular importance [87–89]. In addition, other events in earlier life-history stages, e.g., a delay during breeding, are also shown to influence individual departure decisions [49]. Nevertheless, it is necessary to precisely understand what control mechanisms birds use for optimal migration timing to identify the numerous challenges migratory birds have and will have to face [90] including habitat fragmentation [91], shorter breeding times [92], changes in temperature [93] and habitat alteration or loss [94]. Because of the life-history consequences of poorly timed migratory decisions, it is of critical importance to understand at a population level what mechanism animals use to initiate migration and predict how they will respond to rapid environmental changes in the near future [95].

Recent studies [96, 97] advanced the field of migration on a community level and studied individual flexibility in cues usage [98, 99], yet they lack the combination of individual-level resolution, necessary to determine cue-response relationships, and simultaneous sampling among different species. Studies like the present, using individual tracking data from different populations of the same species, may reveal common control mechanisms and population-specific adaptations, predicting the magnitude of vulnerability to climate change among populations. Future studies should implement common garden experiments or reciprocal translocations to illuminate differences in intrinsic, potentially genetic, cue evaluation or population-specific cue thresholds to expand our knowledge about the control of migration in a period of unprecedented global change.

## Supplementary Information

**Additional file 1:** Tables and figures for additional information on the collected data, environmental conditions and statistical analysis.

## Data Availability

The dataset supporting the conclusions of this article is available from the corresponding authors on reasonable request.

## References

[1] Dingle H. Migration: the biology of life on the move. In: Migration biology of life move. 2nd ed. Oxford: Oxford University Press; 1996.

[2] Winkler DW, Jørgensen C, Both C, Houston AI, McNamara JM, Levey DJ (2014). Cues, strategies, and outcomes: how migrating vertebrates track environmental change. Mov Ecol.

[3] Arnaud CM, Becker PH, Dobson FS, Charmantier A (2013). Canalization of phenology in common terns: genetic and phenotypic variations in spring arrival date. Behav Ecol.

[4] Alerstam T, Hedenström A, Åkesson S (2003). Long-distance migration: evolution and determinants. Oikos.

[5] Nilsson C, Klaassen RHG, Alerstam T (2013). Differences in speed and duration of bird migration between spring and autumn. Am Nat.

[6] Watts HE, Cornelius JM, Fudickar AM, Pérez J, Ramenofsky M (2018). Understanding variation in migratory movements: a mechanistic approach. Gen Comp Endocrinol.

[7] Newton I (2012). Obligater und fakultativer Vogelzug: Ökologische Aspekte. J Ornithol.

[8] Berthold P, Helbig AJ (1992). The genetics of bird migration: stimulus, timing, and direction. Ibis (Lond 1859)..

[9] Smith RJ, Moore FR (2005). Arrival timing and seasonal reproductive performance in a long-distance migratory landbird. Behav Ecol Sociobiol.

[10] Marra PP, Hobson KA, Holmes RT (1998). Linking winter and summer events in a migratory bird by using stable-carbon isotopes. Science.

[11] Gwinner E. Circannual rhythms in bird migration: control of temporal patterns and interactions with photoperiod. Bird Migr. 1990;257–68.

[12] Gwinner E, Helm B. Circannual and circadian contributions to the timing of avian migration. Avian Migr. 2003;81–95.

[13] Gwinner E (1996). Circadian and circannual programmes in avian migration. J Exp Biol.

[14] Newton I (2011). Obligate and facultative migration in birds: ecological aspects. J Ornithol.

[15] Thorup K, Tøttrup AP, Willemoes M, Klaassen RHG, Strandberg R, Vega ML (2017). Resource tracking within and across continents in long-distance bird migrants. Sci Adv.

[16] Watts HE, Robart AR, Chopra JK, Asinas CE, Hahn TP, Ramenofsky M (2017). Seasonal expression of migratory behavior in a facultative migrant, the pine siskin. Behav Ecol Sociobiol.

[17] Mcnamara JM, Barta Z, Klaassen M, Bauer S (2011). Cues and the optimal timing of activities under environmental changes. Ecol Lett.

[18] Åkesson S, Ilieva M, Karagicheva J, Rakhimberdiev E, Tomotani B, Helm B (2017). Timing avian long-distance migration: From internal clock mechanisms to global flights. Philos Trans R Soc B Biol Sci..

[19] Meunier J, Song R, Lutz RS, Andersen DE, Doherty KE, Bruggink JG (2008). Proximate cues for a short-distance migratory species: an application of survival analysis. J Wildl Manag.

[20] Dawson WR, Yacoe ME (1983). Metabolic adjustments of small passerine birds for migration and cold. Am J Physiol Regul Integr Comp Physiol.

[21] Emmenegger T, Hahn S, Bauer S (2014). Individual migration timing of common nightingales is tuned with vegetation and prey phenology at breeding sites. BMC Ecol.

[22] Jarošík V, Honěk A, Magarey RD, Skuhrovec J (2011). Developmental database for phenology models: related insect and mite species have similar thermal requirements. J Econ Entomol.

[23] Eggleton P, Inward K, Smith J, Jones DT, Sherlock E (2009). A six year study of earthworm (Lumbricidae) populations in pasture woodland in southern England shows their responses to soil temperature and soil moisture. Soil Biol Biochem.

[24] Åkesson S, Hedenström A (2000). Wind selectivity of migratory flight departures in birds. Behav Ecol Sociobiol.

[25] Newton I (2007). Weather-related mass-mortality events in migrants. Ibis (Lond 1859).

[26] Metcalfe J, Schmidt KL, Bezner Kerr W, Guglielmo CG, MacDougall-Shackleton SA (2013). White-throated sparrows adjust behaviour in response to manipulations of barometric pressure and temperature. Anim Behav.

[27] Wingfield JC, Pérez JH, Krause JS, Word KR, González-Gómez PL, Lisovski S (2017). How birds cope physiologically and behaviourally with extreme climatic events. Philos Trans R Soc B Biol Sci.

[28] Åkesson S, Hedenstrom A (1996). Flight initiation of nocturnal passerine migrants in relation to celestial orientation conditions at twilight. J Avian Biol.

[29] Åkesson S, Walinder G, Karlsson L, Ehnbom S (2001). Reed warbler orientation: initiation of nocturnal migratory flights in relation to visibility of celestial cues at dusk. Anim Behav.

[30] Ramenofsky M, Cornelius JM, Helm B (2012). Physiological and behavioral responses of migrants to environmental cues. J Ornithol.

[31] Schmaljohann H, Lisovski S, Bairlein F (2017). Flexible reaction norms to environmental variables along the migration route and the significance of stopover duration for total speed of migration in a songbird migrant. Front Zool.

[32] Packmor F, Klinner T, Woodworth BK, Eikenaar C, Schmaljohann H (2020). Stopover departure decisions in songbirds: do long-distance migrants depart earlier and more independently of weather conditions than medium-distance migrants?. Mov Ecol.

[33] Saurola P, Valkama J, Velmala W. The Finnish Bird Ringing Atlas. Vol. I. 2013.

[34] Bairlein F, Dierschke J, Dierschke V, Salewski V, Geiter O, Hüppop K, et al. Atlas des Vogelzugs. Ringfunde deutscher Brut- und Gastvögel. 2014;567.

[35] Pымкeвич TA, Hocкoв ГA, Гaгинcкaя AP, Лaпшин HB, Иoвчeнкo HП, Apтeмьeв AB, et al. Migration of birds of northwest Russia: Passerines. Peнoмe; 2020.

[36] Tomiałojć L, Stawarczyk T. Awifauna Polski: rozmieszenie, liczebność i zmiany. pro Natura; 2003.

[37] Pulido F, Berthold P, Van Noordwijk AJ (1996). Frequency of migrants and migratory activity are genetically correlated in a bird population: evolutionary implications. Proc Natl Acad Sci USA.

[38] Haest B, Hüppop O, Bairlein F (2020). Weather at the winter and stopover areas determines spring migration onset, progress, and advancements in Afro-Palearctic migrant birds. Proc Natl Acad Sci.

[39] Fudickar AM, Schmidt A, Hau M, Quetting M, Partecke J (2013). Female-biased obligate strategies in a partially migratory population. J Anim Ecol.

[40] Newton I. The migration ecology of birds. Migr Ecol Birds. 2007.

[41] Haila Y, Tiainen J, Vepsäläinen K (1986). Delayed autumn migration as an adaptive strategy of birds in northern Europe: evidence from Finland. Ornis Fenn.

[42] Svensson L. Identification guide to European passerines. The author; 1992.

[43] Crofoot MC, Gilby IC, Wikelski MC, Kays RW (2008). Interaction location outweighs the competitive advantage of numerical superiority in *Cebus capucinus* intergroup contests. Proc Natl Acad Sci U S A.

[44] Zúñiga D, Gager Y, Kokko H, Fudickar AM, Schmidt A, Naef-Daenzer B (2017). Migration confers winter survival benefits in a partially migratory songbird. Elife.

[45] Able KP (1997). Control of bird migration Peter Berthold.

[46] R Development Core Team. A language and environment for statistical computing. R Found. Stat. Comput. Vienna: R Foundation for Statistical Computing; 2018. p. https://www.R-project.org.

[47] Meeus JH. Astronomical algorithms. Choice Rev. 1992.

[48] Chatelain M, Halpin CG, Rowe C (2013). Ambient temperature influences birds’ decisions to eat toxic prey. Anim Behav.

[49] Mitchell GW, Newman AEM, Wikelski M, Ryan ND (2012). Timing of breeding carries over to influence migratory departure in a songbird: an automated radiotracking study. J Anim Ecol.

[50] Kalnay E, Kanamitsu M, Kistler R, Collins W, Deaven D, Gandin L (1996). 40-Year reanalysis project. Bull Am Met Soc.

[51] Kemp MU, van Loon EE, Shamoun-Baranes J, Bouten W (2012). RNCEP: global weather and climate data at your fingertips. Methods Ecol Evol.

[52] Glutz von Blotzheim U, Bauer KM (1988). Handbuch der Vögel Mitteleuropas.

[53] Kemp MU, Shamoun-Baranes J, van Loon E, McLaren JD, Dokter AM, Bouten W (2012). Quantifying flow-assistance and implications for movement research. J Theor Biol J Theor Biol.

[54] Healy K (2005). Book review: an R and S-PLUS companion to applied regression. Soc Methods Res.

[55] Benjamini Y, Hochberg Y (1995). Controlling the false discovery rate: a practical and powerful approach to multiple testing. J R Stat Soc Ser B.

[56] Bartlett MS (1937). Properties of sufficiency and statistical tests. Proc R Soc Lond Ser A Math Phys Sci.

[57] Therneau TM, Lumley T (2020). Survival analysis; [R package “survival” version 31–12]. Comp R Arch Netw.

[58] Gienapp P, Hemerik L, Visser ME (2005). A new statistical tool to predict phenology under climate change scenarios. Glob Chang Biol.

[59] Bartoń K. Multi-model inference. 1.43.6. 2019. p. Available at: http://CRAN.R-project.org/package=M.

[60] Guthery FS, Burnham KP, Anderson DR. Model selection and multimodel inference: a practical information-theoretic approach. J. Wildl Manag. 2003.

[61] Hurvich CM, Tsai C-L (1989). Regression and time series model selection in small samples. Biometrika.

[62] Harrison XA, Donaldson L, Correa-Cano ME, Evans J, Fisher DN, Goodwin CED (2018). A brief introduction to mixed effects modelling and multi-model inference in ecology. PeerJ.

[63] Burnham K, Anderson D. Model selection and multimodel inference. A pract information-theoretic approach. 2004.

[64] Kokko H (1999). Competition for early arrival in migratory birds. J Anim Ecol.

[65] Reudink MW, Marra PP, Kyser TK, Boag PT, Langin KM, Ratcliffe LM (2009). Non-breeding season events influence sexual selection in a long-distance migratory bird. Proc R Soc B Biol Sci.

[66] Spottiswoode CN, Tøttrup AP, Coppack T (2006). Sexual selection predicts advancement of avian spring migration in response to climate change. Proc R Soc B Biol Sci.

[67] Nilsson C, Bäckman J, Alerstam T (2014). Seasonal modulation of flight speed among nocturnal passerine migrants: differences between short- and long-distance migrants. Behav Ecol Sociobiol.

[68] Yohannes E, Biebach H, Nikolaus G, Pearson DJ (2009). Migration speeds among eleven species of long-distance migrating passerines across Europe, the desert and eastern Africa. J Avian Biol.

[69] Schmaljohann H (2019). The start of migration correlates with arrival timing, and the total speed of migration increases with migration distance in migratory songbirds: a cross-continental analysis. Mov Ecol.

[70] Brust V, Michalik B, Hüppop O (2019). To cross or not to cross—thrushes at the German North Sea coast adapt flight and routing to wind conditions in autumn. Mov Ecol Movement Ecol.

[71] Panuccio M, Dell’Omo G, Bogliani G, Catoni C, Sapir N (2019). Migrating birds avoid flying through fog and low clouds. Int J Biometeorol.

[72] Liechti F, Bruderer B (1998). The relevance of wind for optimal migration theory. J Avian Biol.

[73] Partecke J, Gwinner E (2007). Increased sedentariness in European blackbirds following urbanization: a consequence of local adaptation?. Ecology.

[74] Schaeffer PJ, Komer MC, Corder KR (2015). Energy savings due to the use of shallow body temperature reduction in overwintering Northern Cardinals. Anim Biotelem.

[75] Gwinner E (1996). Circannual clocks in avian reproduction and migration. Ibis (Lond 1859).

[76] Klaassen M (1996). Metabolic constraints on long-distance migration in birds. J Exp Biol.

[77] Buehler DM, Piersma T (2008). Travelling on a budget: predictions and ecological evidence for bottlenecks in the annual cycle of long-distance migrants. Philos Trans R Soc B Biol Sci.

[78] Piersma T, Jukema J (1990). Budgeting the flight of a long-distance migrant: changes in nutrient reserve levels of bar-tailed godwits at successive spring staging sites. Ardea.

[79] Schmaljohann H, Fox JW, Bairlein F (2012). Phenotypic response to environmental cues, orientation and migration costs in songbirds flying halfway around the world. Anim Behav.

[80] Alerstam T (2011). Optimal bird migration revisited. J Ornithol.

[81] Klaassen RHG, Hake M, Strandberg R, Alerstam T (2011). Geographical and temporal flexibility in the response to crosswinds by migrating raptors. Proc R Soc B Biol Sci Roy Soc.

[82] Alerstam T, Lindström Å. Optimal bird migration: the relative importance of time, energy, and safety. In: Bird Migr. Springer, Berlin; 1990. p. 331–51.

[83] Haest B, Hüppop O, van de Pol M, Bairlein F (2019). Autumn bird migration phenology: a potpourri of wind, precipitation and temperature effects. Glob Change Biol.

[84] Martin GR. The visual problems of nocturnal migration. In: Bird Migr. Springer, Berlin; 1990. p. 185–97.

[85] Åkesson S, Helm B (2020). Endogenous programs and flexibility in bird migration. Front Ecol Evol Front..

[86] Smith AD, McWilliams SR (2014). What to do when stopping over: behavioral decisions of a migrating songbird during stopover are dictated by initial change in their body condition and mediated by key environmental conditions. Behav Ecol.

[87] Eikenaar C, Bairlein F (2014). Food availability and fuel loss predict Zugunruhe. J Ornithol.

[88] Meller K, Vähätalo AV, Hokkanen T, Rintala J, Piha M, Lehikoinen A (2016). Interannual variation and long-term trends in proportions of resident individuals in partially migratory birds. J Anim Ecol.

[89] Studds CE, Marra PP (2011). Rainfall-induced changes in food availability modify the spring departure programme of a migratory bird. Proc R Soc B Biol Sci.

[90] Chapman BB, Brönmark C, Nilsson JÅ, Hansson LA (2011). Partial migration: an introduction. Oikos.

[91] Mac Nally R, Bennett AF, Thomson JR, Radford JQ, Unmack G, Horrocks G (2009). Collapse of an avifauna: climate change appears to exacerbate habitat loss and degradation. Divers Distrib.

[92] Jankowiak Ł, Pietruszewska H, Wysocki D (2014). Weather conditions and breeding season length in blackbird (*Turdus merula*). Folia Zool.

[93] Gatter W (1992). Zugzeiten und Zugmuster im Herbst: Einfluß des Treibhauseffekts auf den Vogelzug?. J Ornithol.

[94] Palomino D, Carrascal LM (2007). Threshold distances to nearby cities and roads influence the bird community of a mosaic landscape. Biol Conserv.

[95] Robertson BA, Rehage JS, Sih A (2013). Ecological novelty and the emergence of evolutionary traps. Trends Ecol Evol.

[96] Doren BMV, Horton KG (2018). A continental system for forecasting bird migration. Science.

[97] O’Neal BJ, Stafford JD, Larkin RP, Michel ES (2018). The effect of weather on the decision to migrate from stopover sites by autumn-migrating ducks. Mov Ecol BioMed Cent.

[98] Burnside RJ, Salliss D, Collar NJ, Dolman PM (2021). Birds use individually consistent temperature cues to time their migration departure. Proc Natl Acad Sci.

[99] Conklin JR, Lisovski S, Battley PF (2021). Advancement in long-distance bird migration through individual plasticity in departure. Nat Commun.

