# Supplementary material for: A partial migrant relies upon a range-wide cue set but uses population-specific weighting for migratory timing
Source: Mov Ecol. 2021 Dec 20;9:63. doi: 10.1186/s40462-021-00298-y (PMC8686659; doi:10.1186/s40462-021-00298-y)
Supplement: Supplementary file 1 — Additional file 1: Tables and figures for additional information on the collected data, environmental conditions and statistical analysis. [file 40462_2021_298_MOESM1_ESM.docx]

Supplementary material

Table S1. Sampled numbers of migrant and resident individuals in four populations of the Eurasian blackbirds (*Turdus merula*). The counts are broken down by sex and age with migrants listed in boldface.

| Population | Strategy | Sex | Age | # Birds |
| --- | --- | --- | --- | --- |
| Germany | **Migratory** | **Female** | **Adult** | **55** |
|  | **Migratory** | **Female** | **Juvenile** | **25** |
|  | **Migratory** | **Male** | **Adult** | **43** |
|  | **Migratory** | **Male** | **Juvenile** | **6** |
|  | **Migratory** | **Unknown** | **Juvenile** | **7** |
|  | Resident | Female | Adult | 114 |
|  | Resident | Female | Juvenile | 13 |
|  | Resident | Male | Adult | 211 |
|  | Resident | Male | Juvenile | 31 |
|  | Resident | Unknown | Juvenile | 22 |
| Finland | **Migratory** | **Female** | **Adult** | **12** |
|  | **Migratory** | **Male** | **Adult** | **14** |
|  | **Migratory** | **Male** | **Juvenile** | **1** |
|  | Resident | Female | Juvenile | 1 |
|  | Resident | Male | Adult | 5 |
|  | Resident | Male | Juvenile | 1 |
| Russia | **Migratory** | **Female** | **Adult** | **7** |
|  | **Migratory** | **Male** | **Adult** | **22** |
|  | **Migratory** | **Male** | **Juvenile** | **1** |
| Poland | **Migratory** | **Female** | **Adult** | **4** |
|  | **Migratory** | **Male** | **Adult** | **15** |

Table S2. Time window of recorded Eurasian blackbird (*Turdus merula*) migration for all included sites and years with corresponding first/last departure (in Julian day) and sample size within specific years. The coefficient of variance for departure is based on all departure dates across all year within each population.

| Site | Year | First departure | Last departure | Length of migration window | Number of Birds | Coefficient of variance for departure |
| --- | --- | --- | --- | --- | --- | --- |
| Finland | 2014 | 289 | 290 | 1 | 3 | 8.3 |
|  | 2015 | 272 | 300 | 28 | 8 |  |
|  | 2016 | 274 | 294 | 20 | 16 |  |
| Germany | 2009 | 262 | 305 | 43 | 14 | 10.6 |
|  | 2010 | 275 | 313 | 38 | 18 |  |
|  | 2011 | 266 | 300 | 34 | 26 |  |
|  | 2012 | 272 | 317 | 45 | 13 |  |
|  | 2013 | 290 | 315 | 25 | 7 |  |
|  | 2014 | 275 | 301 | 26 | 9 |  |
|  | 2015 | 272 | 299 | 27 | 13 |  |
|  | 2016 | 279 | 325 | 46 | 18 |  |
|  | 2017 | 274 | 318 | 44 | 18 |  |
|  | 2018 | 289 | 304 | 15 | 7 |  |
| Poland | 2015 | 270 | 303 | 33 | 11 | 11.4 |
|  | 2016 | 256 | 287 | 31 | 8 |  |
| Russia | 2014 | 261 | 295 | 34 | 13 | 11.1 |
|  | 2015 | 245 | 279 | 34 | 5 |  |
|  | 2016 | 265 | 283 | 18 | 12 |  |

Table S3. Comparison of top time-dependent Cox proportional hazards models to assess the effects of weather variables on birds’ departure probability (night-to-night departure decision). Models’ coefficients and presence of factors are given. Degrees of freedom (df), second-order Akaike’s information criterion values (AICc), AICc differences (Δi) and AICc weights (ωi).

| Model | Residual air temperature | Atmospheric pressure | Crosswind | Day length | Tailwind assistance | Cloud cover | Site x atmos. press. | Site x crosswind | Site x daylength | Site x tailw. assist. | Site x cloud cover | df | logLik | AICc | Δi AICc | weight |
| --- | --- | --- | --- | --- | --- | --- | --- | --- | --- | --- | --- | --- | --- | --- | --- | --- |
| 1 | -0.1856 | 0.282 | 0.624 | -3.492 | 0.236 | -0.470 | + | + | + | + |  | 18 | -811.453 | 1662.3 | 0.00 | 0.368 |
| 2 | -0.1495 | 0.311 | 0.607 | -3.705 | 0.282 | -0.388 | + | + | + | + | + | 21 | -807.828 | 1662.3 | 0.02 | 0.364 |
| 3 |  | 0.291 | 0.535 | -3.784 | 0.358 | -0.406 | + | + | + | + | + | 20 | -809.359 | 1663.0 | 0.63 | 0.268 |

Table S4. Results of the Schoenfeld test for correlations between all used variables in the initial time-dependent Cox proportional hazards model. Chi-squared values (chisq), degrees of freedom (df) and significance level (p)

| Variable | chisq | df | p |
| --- | --- | --- | --- |
| Day length | 2.529 | 1 | 0.11 |
| Residual air temperature | 1.423 | 1 | 0.23 |
| Cloud cover | 1.342 | 1 | 0.25 |
| Atmospheric pressure | 0.044 | 1 | 0.83 |
| Tailwind assistance | 1.348 | 1 | 0.25 |
| Crosswinds | 0.953 | 1 | 0.33 |
| Site x daylength | 3.351 | 3 | 0.34 |
| Site x residual air temperature | 5.298 | 3 | 0.15 |
| Site x cloud cover | 0.759 | 3 | 0.86 |
| Site x atmospheric pressure | 5.496 | 3 | 0.14 |
| Site x tailwind | 6.137 | 3 | 0.11 |
| Site x crosswind | 0.897 | 3 | 0.83 |
| GLOBAL | 30.724 | 24 | 0.16 |

Table S5. Variation in explanatory variables used to explain night-to-night departure decisions in Eurasian blackbirds (*Turdus merula*). Given are the minimum (min.), 10^th^, 25^th^, 50^th^, 75^th^ and 90^th^ percentile as well as the maximum (max.) of each variable and population during nights with departures.

| Population | Variable | Min. | 10^th^  percentile | 25^th^  percentile | Median | 75^th^  percentile | 90^th^  percentile | Max. |
| --- | --- | --- | --- | --- | --- | --- | --- | --- |
| Germany | Day length [h/day] | 9.0 | 10.1 | 10.4 | 10.6 | 10.9 | 11.4 | 12.3 |
|  | Air temperature [°C] | -5.0 | -0.7 | 0.6 | 3.5 | 7.9 | 11.1 | 13.6 |
|  | Atmospheric pressure [mbar] | 913 | 940 | 942 | 948 | 953 | 955 | 959 |
|  | Total cloud cover [%] | 2.4 | 11.3 | 18.8 | 28.1 | 43.3 | 51.4 | 69.6 |
|  | Tailwind assistance [m/s] | -5.3 | -3.3 | -1.6 | 0.3 | 1.5 | 3.3 | 6.7 |
|  | Crosswind [m/s] | -3.1 | -0.5 | 0.0 | 1.0 | 2.4 | 3.5 | 5.1 |
| Finland | Day length [h/day] | 8.9 | 9.6 | 9.8 | 10.5 | 10.9 | 11.3 | 11.5 |
|  | Air temperature [°C] | -3.3 | -1.6 | 0.3 | 1.9 | 3.6 | 7.8 | 12.2 |
|  | Atmospheric pressure [mbar] | 913 | 940 | 942 | 948 | 953 | 955 | 959 |
|  | Total cloud cover [%] | 5.4 | 7.9 | 10.6 | 21.3 | 24.8 | 36.0 | 47.2 |
|  | Tailwind assistance [m/s] | -7.3 | -0.9 | 0.6 | 2.7 | 3.5 | 5.6 | 6.9 |
|  | Crosswind [m/s] | -4.9 | -4.0 | -2.2 | 0.5 | 0.8 | 2.5 | 3.2 |
| Russia | Day length [h/day] | 9.9 | 10.8 | 10.8 | 11.1 | 11.3 | 12.2 | 13.7 |
|  | Air temperature [°C] | -9.6 | 0.8 | 3.5 | 3.6 | 8.6 | 10.7 | 15.1 |
|  | Atmospheric pressure [mbar] | 913 | 940 | 942 | 948 | 953 | 955 | 959 |
|  | Total cloud cover [%] | 1.8 | 10.9 | 11.6 | 32.0 | 53.1 | 81.9 | 81.9 |
|  | Tailwind assistance [m/s] | -3.0 | -0.7 | 1.5 | 2.9 | 7.4 | 7.5 | 7.5 |
|  | Crosswind [m/s] | -6.2 | -3.9 | -2.8 | -0.2 | 1.3 | 1.5 | 2.2 |
| Poland | Day length [h/day] | 9.5 | 10.9 | 11.1 | 11.5 | 11.7 | 11.8 | 12.8 |
|  | Air temperature [°C] | -1.9 | 1.0 | 6.5 | 7.9 | 9.4 | 10.5 | 19.3 |
|  | Atmospheric pressure [mbar] | 913 | 940 | 942 | 948 | 953 | 955 | 959 |
|  | Total cloud cover [%] | 9.7 | 15.6 | 16.4 | 20.6 | 41.1 | 42.7 | 78.8 |
|  | Tailwind assistance [m/s] | -5.3 | -1.3 | 0.6 | 1.2 | 3.7 | 8.1 | 8.1 |
|  | Crosswind [m/s] | -6.3 | -4.8 | -4.6 | -2.5 | -1.7 | 0.5 | 1.4 |

Table S6. Results of the post-hoc pairwise comparison of environmental influence on individual departure decisions between populations of Eurasian blackbirds (*Turdus merula*). Post-hoc tests have been performed on the most complex of the three candidate models (Model 2, Table S3). Provided are adjusted standard errors (SE), z-ratios and associated p-values of pairwise comparisons. P-values ≤ 0.05 are given in bold font.

| Variable | Population contrast | Estimate ± SE | z ratio | p-value |
| --- | --- | --- | --- | --- |
| Day length | Germany - Finland | -0.69 ± 0.55 | -1.25 | 0.25 |
|  | Germany - Russia | -2.09 ± 0.79 | -2.63 | **0.02** |
|  | Germany - Poland | -1.86 ± 0.65 | -2.86 | **0.02** |
|  | Finland - Russia | -1.39 ± 0.54 | -2.57 | **0.02** |
|  | Finland - Poland | -1.17 ± 0.60 | -1.93 | 0.08 |
|  | Russia - Poland | 0.23 ± 0.72 | 0.32 | 0.75 |
| Atmospheric pressure | Germany - Finland | 0.21 ± 0.38 | 0.55 | 0.58 |
|  | Germany - Russia | 0.51 ± 0.33 | 1.55 | 0.18 |
|  | Germany - Poland | -0.95 ± 0.42 | -2.28 | **0.05** |
|  | Finland - Russia | 0.31 ± 0.45 | 0.68 | 0.58 |
|  | Finland - Poland | -1.16 ± 0.53 | -2.18 | 0.06 |
|  | Russia - Poland | -1.46 ± 0.49 | -3.01 | **0.02** |
| Cloud cover | Germany - Finland | -0.39 ± 0.44 | -0.90 | 0.47 |
|  | Germany - Russia | 0.70 ± 0.30 | 2.30 | 0.10 |
|  | Germany - Poland | 0.26 ± 0.45 | 0.58 | 0.56 |
|  | Finland - Russia | 1.09 ± 0.52 | 2.11 | 0.10 |
|  | Finland - Poland | 0.66 ± 0.61 | 1.07 | 0.47 |
|  | Russia - Poland | -0.44 ± 0.51 | -0.86 | 0.47 |
| Tailwind assistance | Germany - Finland | -0.38 ± 0.28 | -1.36 | 0.26 |
|  | Germany - Russia | -1.47 ± 0.41 | -3.56 | **< 0.01** |
|  | Germany - Poland | -0.11 ± 0.28 | -0.39 | 0.69 |
|  | Finland - Russia | -1.10 ± 0.47 | -2.32 | **0.04** |
|  | Finland - Poland | 0.27 ± 0.35 | 0.77 | 0.53 |
|  | Russia - Poland | 1.37 ± 0.47 | 2.94 | **0.01** |
| Crosswind | Germany - Finland | 0.64 ± 0.25 | 2.57 | **0.02** |
|  | Germany - Russia | 0.15 ± 0.34 | 0.43 | 0.67 |
|  | Germany - Poland | 1.29 ± 0.30 | 4.28 | **< 0.01** |
|  | Finland - Russia | -0.50 ± 0.39 | -1.28 | 0.24 |
|  | Finland - Poland | 0.65 ± 0.35 | 1.87 | 0.09 |
|  | Russia - Poland | 1.15 ± 0.42 | 2.70 | **0.02** |


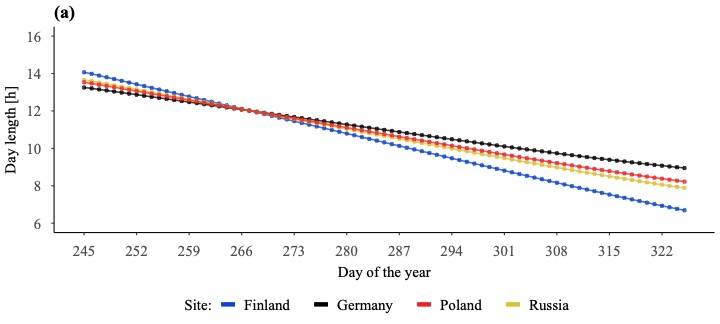

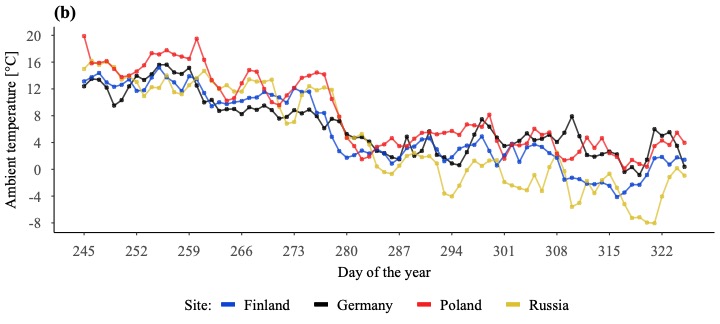

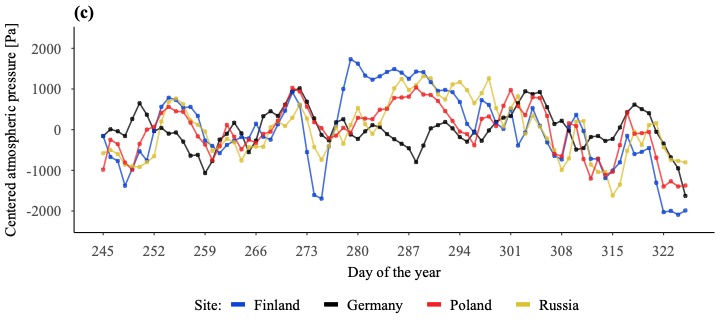

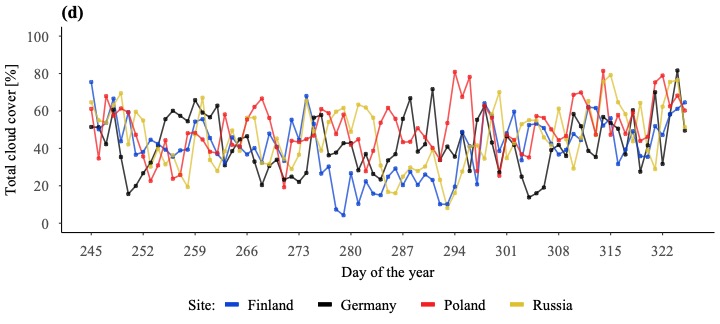

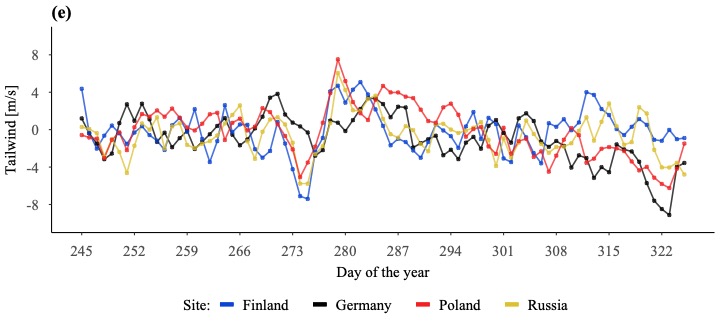

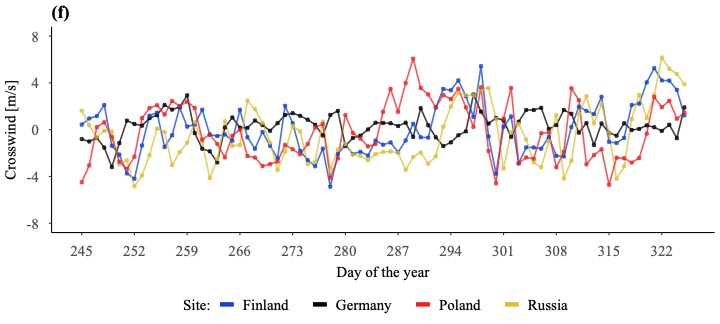


Figure S1a-S1f. **(a)** Daylength, **(b)** Ambient temperature, **(c)** Relative atmospheric pressure, **(d)** Cloud cover , **(e)** Tailwind and **(f)** Crosswind during the overall migration window across all 4 study populations. Shown graphs represent the mean value for each environmental factor across years with observed departures.
